# Barriers and facilitators to use of a clinical evidence technology in the management of skin problems in primary care: insights from mixed methods

**DOI:** 10.5195/jmla.2020.787

**Published:** 2020-07-01

**Authors:** Marianne D. Burke, Liliane B. Savard, Alan S. Rubin, Benjamin Littenberg

**Affiliations:** 1 Marianne.Burke@uvm.edu, Associate Professor of Libraries, Emerita, Dana Medical Library, University of Vermont, Burlington, VT; 2 Liliane.Savard@uvm.edu, Associate Faculty, Rehabilitation and Movement Science, Clinical and Translational Science, University of Vermont, Burlington, VT; 3 Alan.Rubin@med.uvm.edu, Associate Professor, Department of Medicine, University of Vermont, Burlington, VT; 4 Benjamin.Littenberg@med.uvm.edu, Professor of Medicine, General Internal Medicine Research, Larner College of Medicine, University of Vermont, University of Vermont Medical Center, Burlington, VT

## Abstract

**Objective:**

Few studies have examined the impact of a single clinical evidence technology (CET) on provider practice or patient outcomes from the provider's perspective. A previous cluster-randomized controlled trial with patient-reported data tested the effectiveness of a CET (i.e., VisualDx) in improving skin problem outcomes but found no significant effect. The objectives of this follow-up study were to identify barriers and facilitators to the use of the CET from the perspective of primary care providers (PCPs) and to identify reasons why the CET did not affect outcomes in the trial.

**Methods:**

Using a convergent mixed methods design, the authors had PCPs complete a post-trial survey and participate in interviews about using the CET for managing patients' skin problems. Data from both methods were integrated.

**Results:**

PCPs found the CET somewhat easy to use but only occasionally useful. Less experienced PCPs used the CET more frequently. Data from interviews revealed barriers and facilitators at four steps of evidence-based practice: clinical question recognition, information acquisition, appraisal of relevance, and application with patients. Facilitators included uncertainty in dermatology, intention for use, convenience of access, diagnosis and treatment support, and patient communication. Barriers included confidence in dermatology, preference for other sources, interface difficulties, presence of irrelevant information, and lack of decision impact.

**Conclusion:**

PCPs found the CET useful for diagnosis, treatment support, and patient communication. However, the barriers of interface difficulties, irrelevant search results, and preferred use of other sources limited its positive impact on patient skin problem management.

**Figure:**
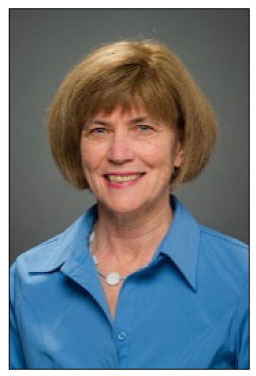
Marianne D. Burke, PhD, AHIP

## INTRODUCTION

Clinical evidence technologies (CETs) are information sources derived from medical research literature that assist health care providers in continued learning, decision making, and patient care. Evidence-based medicine (EBM), defined as “the integration of best research evidence with clinical expertise and patient values” [1], endorses the use of research-based evidence found in CETs—including medical journals, databases, clinical guidelines, and synthesized clinical summaries—to find evidence for patient care. Clinicians report referencing CETs and using the information therein to make better diagnosis and treatment decisions [2–4]. However, they also report barriers to answering their clinical questions, such as poor technology access, lack of relevant evidence sources, and time constraints [5–7].

Dermatology is an area of concern in primary care for which previous literature extensively discusses the goals of improving diagnostic accuracy, improving the management of skin disease, and reducing referrals [8–12]. Some studies in primary care and hospital settings show that a dermatology education tool kit [13] and diagnostic support CET [14] can improve provider confidence and diagnostic accuracy. However, few studies have examined the impact of a single CET on provider practice or patient outcomes from the provider's perspective.

The present study followed up on a previous cluster-randomized controlled trial to understand why and how primary care providers (PCPs) used a CET, VisualDx, to care for patients with skin disease [15]. VisualDx, a factual knowledge database and diagnostic tool, matches patient symptoms with images to suggest likely diagnoses and management strategies [16]. In the original trial, 32 PCPs were randomly assigned to use or not use VisualDx, and over 400 of their patients with skin complaints were interviewed about the outcomes of their primary care visits. PCP participation in the original trial averaged 6 months. Study results showed that VisualDx use did not have a significant effect on the resolution of symptoms or the number of return appointments.

The objectives of this follow-up investigation were twofold: (1) to identify barriers and facilitators to PCPs' use of the CET in a patient care context and (2) to gain insight from PCP reports into why CET use did not affect patient-level outcomes.

## METHODS

The authors used a convergent, mixed methods design [17], in which we combined a quantitative survey with qualitative interviews to realize a more complete understanding of PCPs' experiences using the CET in a complex patient care setting. The methods had equal priority and were conducted concurrently in February and March of 2018, nineteen to twenty months after PCPs' participation in the original trial had concluded. We followed the guidelines of O'Cathain et al. for reporting mixed methods to enhance the clarity of the methodology and analysis presentation [18]. The University of Vermont Institutional Review Board approved the original clinical trial, including baseline and post-surveys of PCPs in May 2015 and the qualitative interview investigation in January 2018.

Participants included faculty and residents in family medicine and internal medicine primary care clinics who participated in the original trial. All PCPs in the original trial were invited to participate in the closed-answer post-trial survey, which was administered online or in-person (supplemental Appendix A). Data were entered and stored in REDCap [19]. Demographic data—including years in practice, resident versus attending status, family medicine versus internal medicine status, and gender—were collected in the baseline survey of the original trial. Questions in the post-trial survey varied by participant arm. All PCPs were queried on their use of VisualDx during and after the trial and their use of other information sources after the trial. PCPs in the CET arm were also asked about the number of times used, ease of use, and usefulness of VisualDx. The survey instrument design was informed by the technology acceptance model, which posits that intention, perceived ease of use, and perceived usefulness are important factors for acceptance and continued use of technologies introduced in the workplace [20]. Survey data were analyzed with descriptive statistics in Stata version 14.2 [21].

**Figure 1:**
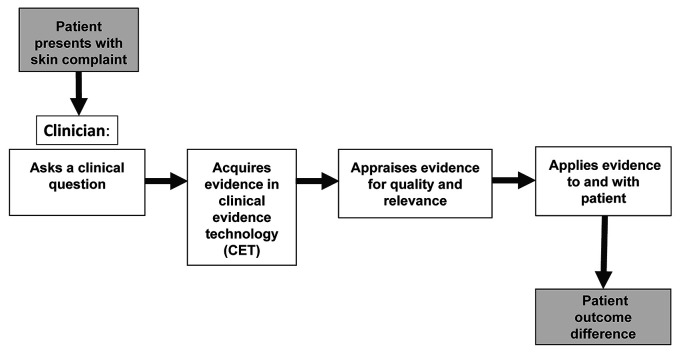
Behavioral steps model based on the evidence-based medicine (EBM) paradigm

PCPs in the CET arm also participated in semi-structured interviews conducted in-person and digitally recorded by the principal investigator (PI) (supplemental Appendix B). Interviews were transcribed by the PI and a research assistant. We chose a behavioral steps model based on the EBM paradigm to inform the semi-structured interview instrument design and to frame the analysis of qualitative data (Figure 1). The EBM paradigm includes sequential behavioral steps that clinicians took to find and apply the best available evidence. These steps, as described in EBM textbooks [1, 22] and reaffirmed by expert teaching and clinician panels [23], are: (1) ask clinical questions when uncertainty arises, (2) acquire the best available evidence, (3) appraise and interpret the evidence found for quality and relevance, and (4) apply evidence considering patient values and preferences [22, 23]. The PI conducted initial coding of PCP statements using NVivo version 12 qualitative analysis software [24]. The PI and two independent team members then refined codes and identified emergent themes. Final themes were decided by team consensus. We organized themes as barriers or facilitators and noted when PCPs referenced the themes at each behavioral step.

To integrate the quantitative and qualitative results, we compared survey results relating to ease of use and usefulness to interview themes, using the triangulation protocol described by O'Cathain et al. [25], which utilizes concepts of convergence, complementarity, dissonance, and silence to compare findings between methods in mixed methods studies.

## RESULTS

### Quantitative survey results

Twenty-one of the 32 (66%) PCPs in the original trial participated in the post-trial survey: 13 of 17 (76%) in the CET arm, and 8 of 15 (53%) in the control arm (Table 1).

**Table 1:** Characteristics of primary care providers (PCPs) and clinical evidence technology (CET) usage in post-trial survey

	All (n=21)		Clinical evidence technology (CET) (n=13)		Control (n=8)	
	n	(%)	n	(%)	n	(%)
Gender (men)	10	(48%)	6	(46%)	4	(50%)
Resident (vs. Attending)	4	(19%)	4	(31%)	0	—
Family medicine (vs. Internal medicine)	10	(48%)	5	(38%)	5	(63%)
Primary care providers (PCPs) education						
Physician	20	(95%)	13	(100%)	7	(88%)
Advanced practice nurse	1	(5%)	0	(—)	1	(13%)
Followed VisualDx usage protocol in the trial	20	(95%)	13	(100%)	7	(88%)
Used VisualDx after the trial (yes)	14	(67%)	9	(69%)	5	(63%)
Years in practice						
Median	17		12		18	
Range	1–40		1–40		2–39	
Times used VisualDx during the trial						
Median	10					
Range	3–125					

#### Protocol fidelity and frequency.

PCPs in the CET arm used VisualDx during the trial, whereas PCPs in the control arm, with 1 exception, did not, indicating protocol fidelity in both arms. PCPs in the CET arm used VisualDx a median of 10 times in the 6-month average trial participation period. Nearly half of PCPs in the CET arm (46%) reported using VisualDx with most of their patients with skin problems.

#### Ease of use and usefulness.

Of the PCPs in the CET arm, 10 (77%) described VisualDx as “somewhat easy” or “very easy” to use, whereas 3 (23%) found it “somewhat difficult” or “difficult” to use. When asked if VisualDx was useful for diagnosing and treating patients, 5 PCPs (38%) responded “usually,” 5 (38%) responded occasionally, and 3 (23%) responded “not at all”; none found it “always” useful. These findings indicated that the CET was perceived as easier to use than actually useful (Table 2).

**Table 2:** CET frequency of use, ease of use, and usefulness, depending on years in practice

	All CET users (n=13)		Practice years ≤5 (n=6)		Practice years >5 (n=7)	
	n	(%)	n	(%)	n	(%)
VisualDx use during the trial, median uses	10	range: 3–125	15	range: 5–30	10	range: 3–125
Used VisualDx with >50% of skin patients	6	(46%)	4	(67%)	2	(29%)
Ease of use						
Very or somewhat difficult	3	(23%)	0	(—)	3	(43%)
Very or somewhat easy	10	(77%)	6	(100%)	4	(57%)
Usefulness						
Not at all or occasionally useful	8	(62%)	3	(50%)	5	(71%)
Usually or always useful	5	(38%)	3	(50%)	2	(29%)

#### Years in practice.

Compared with more experienced PCPs, PCPs with 5 or fewer years in practice used the CET more often (median 10 versus 15 times) and were more likely to use the CET with more than half of their patients (67% versus 29%). All (100%) of less experienced PCPs found the CET very or somewhat easy to use (100%), compared with 57% of more experienced PCPs (Table 2).

#### Usage of VisualDx and other CETs post-trial.

Two-thirds (67%) of the 21 PCPs used VisualDx after the trial, and all (100%) used other information sources for the care of patients with skin problems. In a typical month post-trial, 6 PCPs (29%) reported using VisualDx, 11 (52%) used UpToDate, 6 used textbooks (29%), 4 used Google (19%), 1 used Epocrates (5%), and 1 used DynaMed (5%). None used PubMed/MEDLINE, other citation databases, or journal articles.

### Qualitative interview results

Eleven PCPs in the CET arm participated in an interview, including three residents and eight attending physicians who had been in practice for one to forty years. We organized PCP interview statements into facilitator and barrier themes and noted the behavioral step context of the statement. Facilitator themes included intention to use the CET, uncertainty in dermatology, electronic health record (EHR) access, diagnosis or treatment support, and patient communication. Barrier themes included confidence in dermatology, time pressure, interface difficulties, use of other preferred sources, irrelevant information, and lack of impact on patient care. Facilitators and barriers to use of the CET at each behavioral step of the EBM model, with representative PCP statements are presented in Table 3.

**Table 3:** Representative PCP statements related to facilitators and barriers to CET use aligned with behavioral evidence-based medicine (EBM) steps

Facilitator or barrier	Theme	Provider statements
Step 1: Ask clinical questions when uncertainty arises		
Facilitators	Intention to use CET	“I think I used it close to every time I saw a skin problem, unless it was super obvious…But even then, I would use it to get treatment recommendations.” PCP08 (Resident, 3 years) “When I had a patient that had a skin complaint, I was supposed to open VisualDx…I tried to be pretty diligent about it.” PCP01 (Resident, 1 year)
	Uncertainty in dermatology	“ [Dermatology] is way harder because we just don't have the exposure. So, I think something like VisualDx is totally necessary.” PCP07 (Resident, 3 years) “There are certain areas, [like dermatology] where internists in particular, don't have as much training and we tend to fall into…less rigorous ways of approaching a diagnosis.” PCP10 (Attending, 22 years)
Barriers	Confidence in dermatology	“If it's a simple thing that. you feel like you know what it is and how to treat it, then you obviously wouldn't use the resource in that situation.” PCP02 (Attending, 32 years) “There were a lot of patients where I felt comfortable with what the problem was.” PCP11 (Attending, 24 years)
	Other preferred information sources	“I was working.next to a skilled, older practitioner. So often times my first recourse would be going to him.” PCP09 (Attending, 4 years) “I used UpToDate quite frequently. And I used Micromedex quite frequently.. don't think my use of VisualDx changed my rates of use of those other resources.” PCP08 (Resident, 3 years) “I have a favorite dermatology book that I use like I would use VisualDx.” PCP10 (Attending, 22 years) “Sometimes I just used Google Images.” PCP09 (Attending, 4 years)
	Time pressure	“When you are already 45 minutes behind schedule and someone comes in with an [odd] rash, 'It's easy to say, I think it's this, try it, if it doesn't work call me back.'” PCP10 (Attending, 22 years)
Step 2: Acquire the best available evidence		
Facilitators	Electronic health record (EHR) access	“If I'm seeing patients, I'm already in the [electronic medical record] EMR, and VisualDx is there. It's easy to find. 99% of the time that's what I'd do.” PCP11 (Attending, 24 years)
	CET interface	“Once I knew what I was doing it, it wasn't hard to use.” PCP06 (Attending, 4 years)
Barrier	CET interface	“I remember staring at it saying, 'Where do I put the information in?' So, it wasn't as user friendly for data input.” PCP10 (Attending, 22 years) “I'm not sure if I'm just not putting in enough [information].” PCP09 (Attending, 4 years)
Step 3: Appraise and interpret the evidence found for quality and relevance		
Facilitators	Quality of evidence	“I had a lot of confidence that the material was accurate and properly edited or authenticated by experts in the field.” PCP03 (Attending, 34 years) “The problem with Google Images is [that] anybody…can upload a picture and tag it with a diagnosis.” PCP10 (Attending, 22 years)
	Diagnosis support	“I did, on a few occasions have no idea what I was looking at in a patient, and used [VisualDx]…to figure it out.” PCP08 (Resident, 3 years) “I can definitely say it helped me feel more confident about a diagnosis.” PCP02 (Attending, 32 years) “I would often look at a skin lesion or rash and have an idea. and then. VisualDx would broaden my differential and sometimes completely change my initial opinion.” PCP07 (Resident, 3 years)
	Treatment support	“A lady came in with something strange on her eyes. Based on using VisualDx I came up with something I hadn't considered. That did prompt a referral to dermatology.” PCP01 (Resident, 1 year) “I think it changed my rate of dermatology referrals because I [was] willing to diagnose skin conditions with.more confidence and to act on those diagnoses.” PCP08 (Resident, 3 years)
Barriers	Presence of irrelevant information	“Just as frequently as I found that it was helpful, I found that it was not helpful at all…I mostly got a lot of extraneous information and things that…weren't appropriate for what I was looking for. So some of that time using it was wasted.” PCP08 (Resident, 3 years) “If you put basal cell carcinoma in VisualDx, it's a thousand pictures of every possible way it can show up. It's not showing the typical ones.” PCP03 (Attending, 34 years) “I remember getting more hits back…a lot more diagnoses — than I was expecting — some of which didn't even look close to what I described.” PCP10 (Attending, 22 years)
	Other preferred information sources	“If I knew what the [diagnosis] was but didn't know how to manage it, I might use UpToDate [more].” PCP11 (Attending, 24 years) “If I thought of something, I'd look it up on UpToDate [also] and see if the pictures and descriptions matched [VisualDx]” PCP06 (Attending, 4 years)
Step 4: Apply evidence considering patient values and preferences		
Facilitators	Patient communication	“I used it with patients, especially if they had something that went away; then they could say, 'Oh, it did look like that.'” PCP04 (Attending, 17 years) “Helpful for patient communication? Absolutely.” PCP04 (Attending, 17 years) “If you can use a visual to show somebody and say, 'Oh this looks like really what you have,' they gain a little bit more confidence.” PCP09 (Attending, 4 years)
	Shared decision making	“I would open it up in the patient room oftentimes, and go through it [all] with them.” PCP06 (Attending, 4 years) “I would look at VisualDx and it would give me additional ideas. So, then I would talk to the patient more, come up with a diagnosis.” PCP08 (Resident, 3 years)
Barrier	No impact on patient care	“I can't think of a particular instance where it clinched it for me or made a clinical decision distinction or difference. It was more of a tool that I used to augment whatever I was looking into.” PCP09 (Attending, 4 years) “Care difference? I would have to say no, that it didn't really offer me a different path forward.” PCP03 (Attending, 34 years) “If I was going to refer to dermatology, I [would] refer to dermatology. [VisualDx] wouldn't change my mind.” PCP04 (Attending, 17 years)

#### Step 1: Ask clinical questions when uncertainty arises.

Facilitators to using the CET at this step were uncertainty in dermatology and intention to use the CET with skin problem patients. Some PCPs recognized uncertainty in dermatology, especially the diagnosis of rashes, as an area of concern due to less training and fewer rigorous approaches than in other domains. Several residents stated that because of such uncertainty, evidence-based information resources in dermatology were especially needed. One resident expressed her intention to use the CET from the beginning and estimated that she used it with nearly all her patients with skin problems.

Barriers to using the CET at this step were confidence in dermatology, use of other preferred sources, and time pressure. PCPs who expressed confidence felt less need for information seeking. Some had taken additional course work in dermatology, which increased their confidence and reduced their CET use. Use of other evidence sources instead of VisualDx also deterred CET use. Some did not always choose VisualDx as their first or only source, despite the trial protocol. Instead, PCPs felt that colleagues and other CETs—such as print textbooks, UpToDate, drug databases, and Internet images—would be better at times. Furthermore, perceived lack of time in a patient encounter prevented PCPs from seeking answers from any information source, even when they recognized uncertainty. Instead, they sometimes used a “try this and see if it works” approach.

#### Step 2: Acquire the best available evidence.

Facilitators to using the CET at this step were access to the CET through the EHR and perceived overall ease of use of the CET. The EHR was almost always the only means by which PCPs accessed VisualDx, as it was convenient to access quickly from the desktop computers in patient exam rooms. Although the CET mobile version was available on smart phones and tablets, PCPs did not use it for patient care. Several stated they found the VisualDx interface easy to learn and use, though there was a “small learning curve.”

Despite its overall ease of use, the main barrier to CET use at this step was interface difficulties. About half of PCPs found the CET's interactive diagnosis tool confusing, “not user friendly,” and unpredictable. Some lacked confidence in their ability to use the CET effectively, even though they viewed a training tutorial as part of their enrollment in the trial. One PCP reported she lost access to the CET through the EHR after one month in the trial and did not return to using the resource, even though she received assistance from a technical help desk.

#### Step 3: Appraise and interpret the evidence found for quality and relevance.

Facilitators to using the CET at this step were the availability of good quality evidence, assistance in patient diagnosis, and treatment decision support. PCPs appraised VisualDx information as good and reliable because it was validated by expert dermatologists. They knew it was more reliable than images found via Internet search engines, to which “anybody…can upload a picture.” None described seeking higher levels of evidence, such as diagnostic tools that had been evaluated in randomized trials usually found in the journal literature. The CET's relevance to diagnosis emerged to support differential diagnosis expansion and confirmation of diagnosis. Residents found the CET's interactive diagnostic tool particularly relevant when they had little idea of the diagnosis and needed to broaden the differential. Experienced physicians more often wanted to confirm a diagnosis, which VisualDx supported at times. With confirmation, PCPs were more likely to treat the problem themselves and avoid a referral. There were also situations in which diagnosis confirmation prompted a referral. Furthermore, new treatments described in the CET affected some PCPs' treatment decisions and served to update their usual practice.

Barriers to using the CET at this step included the presence of irrelevant information. PCPs often retrieved too much information, which required time-consuming information sifting or a new search. Experienced clinicians, in particular, felt that the range of diagnoses and images that the CET retrieved was excessively broad, making it difficult to narrow the differential or confirm the most likely diagnosis. In addition, PCPs considered the CET to be one information source among others to assist with the management of skin conditions, even though other sources were not optimized for this topic. VisualDx was used as “just one tool” among others or as a corroborator of evidence that was found in another source.

#### Step 4: Apply evidence considering patient values and preferences.

Facilitators to using the CET at this step were patient communication and shared decisions. PCPs found VisualDx images and information applicable for educating patients and building rapport. The images helped them show patients how their conditions had improved, which enhanced agreement on treatments and patients' confidence. A few PCPs found the dermatology images too graphic to show patients but did share the information that they found. Some shared a full range of VisualDx information with patients, including alternative diagnoses and multiple images, in a shared decision-making process.

The main barrier to using the CET at this step was a lack of found evidence that applied to a particular patient. Despite positive examples of communication with patients, many PCPs did not recall any real impact of using the CET with patients. That is, the information retrieved was relevant in a general way but did not aid in making decisions or offering a “different path forward” from what the PCP would have done anyway.

### Mixed methods results integration

When combined, the quantitative survey and qualitative interview results provide a more complete picture of how PCPs sought and used VisualDx and other information sources to manage patients' skin problems. The interviews provided context related to each behavioral step of EBM for the survey responses pertaining to frequency of use, ease of use, and usefulness for patient care and identified specific barriers and facilitators to CET use. When we compared four survey variables (usage of the CET, ease of use, usefulness, and use of other information sources) with the interview themes and subthemes at the behavioral steps, most comparisons reflected complementarity, such that the interview statements did not contradict but rather expanded upon the survey responses (Table 4).

**Table 4:** Integration of mixed methods

Behavioral step	Survey results	Triangulation	Interview results: barriers (B) and facilitators (F)
Step 1: Ask clinical questions when uncertainty arises	PCPs used the CET a median of 10 times; less experienced PCPs used the CET a median of 15 times.	Complementarity	PCPs expressed intention and frequent usage (F)
	46% of PCPs used the CET with most patients.	Complementarity	Experienced PCPs who expressed confidence in dermatology also expressed a lack of need and lower usage (B), whereas uncertainty signaled more need and usage (F).
Step 2: Acquire the best available evidence	77% of PCPs found the CET somewhat or very easy to use.	Convergence	All but 1 PCP found CET access through the EHR to be easy (F). The CET interface was easy to use for about half of PCPs (F).
	No data on CET interface or EHR aspects.	Partial silence	About half of PCPs reported that the interactive diagnosis tool was difficult and unpredictable at times (B).
Step 3: Appraise and interpret the evidence found for quality and relevance	No data on evidence quality.	Silence	PCPs expressed that the quality of evidence in the CET was satisfactory (F).
	62% of PCPs reported that the CET was not useful or occasionally useful for diagnosis and treatment, whereas 38% reported that it was usually useful.	Complementarity	PCPs expressed that the CET was relevant and useful for differential diagnosis expansion, diagnosis confirmation, and treatment discovery (F). Others said it was “just as often” irrelevant or unhelpful (B).
	67% of PCPs used VisualDx in a recent month post-trial.	Complementarity	PCPs reported that other information sources were as or more useful than the CET (B).
Step 4: Apply evidence considering patient values and preferences	No specific data on application to patients.	Silence	PCPs expressed that the CET facilitated patient education and shared decisions (F), and prompted and avoided referrals (F), but had little application to specific patient decisions (B).

## DISCUSSION

This study identified facilitators and barriers to effective use of a dermatology-focused CET for skin problem management in the context of patient care from the perspective of PCPs. It also identified possible reasons why use of the CET did not impact patient outcomes in the original trial. The brief closed-answer survey of PCPs provided summary information on the number of times used, ease of use, and usefulness of the CET. Barriers and facilitators identified in interviews enriched our understanding of the complex behavioral EBM steps that influenced use of a CET. Integration of the results of mixed methods provided complementary insights.

### Barriers and facilitators to CET use in evidence-based practice

Multiple barriers to the use of clinical evidence sources by PCPs have been described in the literature over the last decades. In two studies of PCPs, Ely et al. identified lack of time to seek and acquire needed information and lack of skill as barriers [5, 26]. Ely et al. also identified the retrieval of too much information and the irrelevance of the retrieved information as problems. In a focus group study of primary care internal medicine residents, poor access to technology and lack of relevant sources in the practice setting were barriers [7]. These same barriers were also identified in our study. In addition, a qualitative study identified the barrier of failure of the evidence sources to account for patient complexity [27]. A 2012 systematic review including twenty-two studies published between 1997 and 2010 reported barriers to EBM practice at each behavioral step that were similar to those reported in previous literature, except for a novel barrier at the Apply Evidence step: patient disagreement with the best evidence [28]. An additional barrier identified in our study was PCP confidence in the dermatology domain.

Cook et al. identified multiple facilitators in a study that identified strengths of “knowledge resources” (i.e., CETs). Effective sources were found to be efficient, credible, integrated with the clinical workflow, familiar to the user, optimized for the topic, and supportive of patient education [29]. In our study, convenient access to VisualDx through the EHR partially overcame the barrier of time pressure. The ability to include patient factors in the interactive diagnosis tool partially accounted for relevance to complex patient characteristics. However, the unpredictability of search results decreased efficiency and reduced the benefit of using VisualDx as opposed to other familiar sources. Utility for patient communication, education, and shared decision making emerged as a benefit when evidence was applied with patient preferences and values, an essential step in evidence-based practice.

Seeking information from multiple sources for the same clinical question is typical behavior for clinicians [2, 30]. One study noted that 3.5 CET sources were typically referenced per question [30]. In our study, PCPs preferred multiple CETs, if they were convenient. The presence of other sources diluted the impact of VisualDx and reduced the likelihood of detecting any effects of CET use on patient outcomes in the original trial.

It is possible that evidence seeking by CETs may be less frequent or more difficult in dermatology. In a qualitative study of PCPs' strategies for diagnosing skin problems, their preferred strategies included pattern recognition, “trying out” treatments, and referral to dermatology. Consulting research-based literature or online sources was seldom used as a strategy [31]. In our study, nearly half of PCPs in the CET arm reported using VisualDx with most of their patients with skin problems, and they frequently used other evidence sources if they were convenient. This study did not identify any dermatology evidence source as superior to VisualDx, only that PCPs used it among other CETs for management of dermatological problems.

Our results suggested that VisualDx might be more useful to trainees and new attending PCPs than those with more experience. Less experienced PCPs seemed to express more ease using the CET, recognized more uncertainty in dermatology, and expressed the need for tools like VisualDx. For these users, expansion of the differential diagnosis with use of the patient-specific interactive diagnosis tool facilitated point-of-care learning.

### Effect on patient-level outcomes

Why did VisualDx use make no difference in the outcomes reported in the original study? It was possible that the effects were bidirectional. For instance, some PCPs reported that VisualDx use affected their referral patterns. For some, the evidence found for a diagnosis prompted referrals to dermatology. For others, a referral was avoided, and the clinician gained confidence in treating the condition. This effect might partially explain why use of the CET did not reduce the overall number of patient return appointments (including referrals) for the same skin problem (odds ratio=1.26, 95% confidence interval=0.70–1.21, *p*=0.54) [15].

Likewise, three other intervention studies found that use of a CET did not reduce referrals to dermatology [13, 32, 33]. While reduction of referrals and other return appointments may be a clinical goal to save patient and provider time and to reduce costs, its attainment through usage of CETs has not been established. It is possible that patient communication while using the CET could have affected patient satisfaction with care, which could be evaluated in future research.

### Implications for evaluation of CETs

Although this study focused on one CET, the barriers and facilitators to its use might be applicable in the evaluation of other CETs implemented for point-of-care use. We identified ways that a single CET may have value for providers' management of patient conditions, such as diagnostic accuracy and identification of best treatments. A CET may also facilitate point-of-care learning and shared decision making with patients.

Health sciences librarians directly support the Acquire Evidence step in the EBM model by licensing and providing access to clinical evidence sources. When choosing and licensing CETs, medical librarians should consider the factors of clinician population, access to technology, and available evidence sources in addition to cost. A CET licensed and implemented for clinical use should be accessible through the EHR to increase clinician acceptance. Less experienced clinicians and residents may have different CET use patterns than more experienced PCPs. Furthermore, use of more sources may be needed to meet clinicians' clinical evidence needs for the care of skin problems.

### Limitations

Our study had several limitations that should be considered. It did not include reports from patients, limiting the interpretations to the perceptions and experience of PCPs. Recall errors might have affected the reported data, but all PCPs appeared to respond to survey and interview questions without difficulty. The interviews were conducted by a medical librarian known to some of the PCPs outside the study, which could have introduced bias. However, all PCPs agreed to give their true opinions and were assured that their responses would be confidential and would not affect their access to medical library services. In addition, the study took place in one academic medical center, limiting its generalizability to other settings.

## CONCLUSION

We identified facilitators and barriers to PCPs' use of a CET for skin problems in the context of patient care, which partially explains the results of a previous cluster-randomized controlled trial. We found that the CET was not consistently useful to PCPs or applicable to patients. However, it did support some diagnosis and treatment decisions, point-of-care learning, and patient communication and shared decision making. These findings could be useful to clinical administrators and medical librarians who are considering implementing CETs to support the management of dermatological conditions in primary care settings.

## Data Availability

Data supporting the findings of this study are openly available at https://figshare.com/articles/Use_Skin_CET_survey_dataset_csv/11893875 for quantitative survey data, and https://figshare.com/articles/Use_of_a_Clinical_Evidence_Technology_for_Skin_Disease_in_Primary_Care_Clinician_Interviews/11893956 for qualitative interview transcripts.

## SUPPLEMENTAL FILES

**Appendix A:** Survey of primary care providers post-cluster-randomized controlled trial

**Appendix B:** Semi-structured interviews outline

## References

[1] Sackett DL Evidence-based medicine: how to practice and teach EBM. London, UK: Churchill Livingstone; 2000.

[2] Marshall JG, Sollenberger J, Easterby-Gannett S, Morgan LK, Klem ML, Cavanaugh SK, Oliver KB, Thompson CA, Romanosky N, Hunter S The value of library and information services in patient care: results of a multisite study. J Med Libr Assoc. 2013 1;101(1):38–46. DOI: 10.3163/1536-5050.101.1.007. 23418404 PMC3543128

[3] Sievert M, Burhans D, Ward D, Jones BB, Bandy M, Carlson J, Decker S, Henderson H Value of health sciences library resources and services to health care providers in medium and large communities across two Mid-Continental states. J Hosp Librariansh. 2011;11(2):140–57. DOI: 10.1080/15323269.2011.558882.

[4] Alper BS, White DS, Ge B Physicians answer more clinical questions and change clinical decisions more often with synthesized evidence. Ann Fam Med. 2005 11;3(6):507–13. DOI: 10.1370/afm.370. 16338914 PMC1466938

[5] Ely JW, Osheroff JA, Chambliss ML, Ebell MH, Rosenbaum ME Answering physicians' clinical questions: obstacles and potential solutions. J Am Med Inform Assoc. 2005 Mar-Apr;12(2):217–24. DOI: 10.1197/jamia.M1608. 15561792 PMC551553

[6] Andrews JE, Pearce KA, Ireson C, Love MM Information-seeking behaviors of practitioners in a primary care practice-based research network (PBRN). J Med Libr Assoc. 2005 4;93(2):206–12. 15858623 PMC1082937

[7] Green ML, Ruff TR Why do residents fail to answer their clinical questions? a qualitative study of barriers to practicing evidence-based medicine. Acad Med. 2005 2;80(2):176–82. 15671325 10.1097/00001888-200502000-00016

[8] Federman DG, Reid M, Feldman SR, Greenhoe J, Kirsner RS The primary care provider and the care of skin disease: the patient's perspective. Arch Dermatol. 2001 1;137(1):25–9. 11176657 10.1001/archderm.137.1.25

[9] Fleischer AB Jr., Herbert CR, Feldman SR, O'Brien F Diagnosis of skin disease by nondermatologists. Am J Manag Care. 2000 10;6(10):1149–56. 11184670

[10] Verhoeven EW, Kraaimaat FW, van Weel C, van de Kerkhof PC, Duller P, van der Valk PG, van den Hoogen HJ, Bor JH, Schers HJ, Evers AW Skin diseases in family medicine: prevalence and health care use. Ann Fam Med. 2008 Jul-Aug;6(4):349–54. DOI: 10.1370/afm.861. 18626035 PMC2478509

[11] Ojeda RM, Graells J [Effectiveness of primary care physicians and dermatologists in the diagnosis of skin cancer: a comparative study in the same geographic area]. Actas Dermosifiliogr. 2011 1;102(1):48–52. DOI: 10.1016/j.ad.2010.06.020. 21315861

[12] Kownacki S Skin diseases in primary care: what should GPs be doing? Br J Gen Pract. 2014 8;64(625):380–1. DOI: 10.3399/bjgp14X680773. 25071029 PMC4111309

[13] Gulati A, Harwood CA, Rolph J, Pottinger E, McGregor JM, Goad N, Proby CM Is an online skin cancer toolkit an effective way to educate primary care physicians about skin cancer diagnosis and referral? J Eur Acad Dermatol Venereol. 2015 11;29(11):2152–9. DOI: 10.1111/jdv.13167. 25917519

[14] David CV, Chira S, Eells SJ, Ladrigan M, Papier A, Miller LG, Craft N Diagnostic accuracy in patients admitted to hospitals with cellulitis. Dermatol Online J. 2011 3 15;17(3):1. 21426867

[15] Burke M, Littenberg B Effect of a clinical evidence technology on patient skin disease outcomes in primary care: a cluster-randomized controlled trial. J Med Libr Assoc. 2019 4;107(2):151–62. DOI: 10.5195/jmla.2019.581. 31019383 PMC6466492

[16] Logical Images. VisualDx [Internet]. Logical Images [cited 22 Oct 2018]. <https://www.visualdx.com/visualdx/7/>.

[17] Creswell JW Chapter 10: Mixed methods procedures. In: Creswell JW Research design: qualitative, quantitative, and mixed methods approaches. 4th ed. Thousand Oaks, CA: SAGE Publications; 2014 pp. 215–40.

[18] O'Cathain A, Murphy E, Nicholl J The quality of mixed methods studies in health services research. J Health Serv Res Policy. 2008 4;13(2):92–8. DOI: 10.1258/jhsrp.2007.007074. 18416914

[19] Harris PA, Taylor R, Thielke R, Payne J, Gonzalez N, Conde JG Research electronic data capture (REDCap)—a metadata-driven methodology and workflow process for providing translational research informatics support. J Biomed Inform. 2009 4;42(2):377–81. DOI: 10.1016/j.jbi.2008.08.010. 18929686 PMC2700030

[20] Davis FD Perceived usefulness, perceived ease of use, and user acceptance of information technology. MIS Q. 1989 9;13(3):319–40. DOI: 10.2307/249008.

[21] StataCorp. Stata statistical software: release 14. College Station, TX: StataCorp; 2015.

[22] Straus SE Evidence-based medicine: how to practice and teach it. 4th ed. Edinburgh, UK: Elsevier Churchill Livingstone; 2011 293 p.

[23] Albarqouni L, Hoffmann T, Straus S, Olsen NR, Young T, Ilic D, Shaneyfelt T, Haynes RB, Guyatt G, Glasziou P Core competencies in evidence-based practice for health professionals: consensus statement based on a systematic review and delphi survey. JAMA Network Open. 2018 6 1;1(2):e180281 DOI: 10.1001/jamanetworkopen.2018.0281. 30646073

[24] QSR International. NVivo qualitative data analysis software version. 12th ed. QSR International; 2018.

[25] O'Cathain A, Murphy E, Nicholl J Three techniques for integrating data in mixed methods studies. BMJ. 2010 9 17;341:c4587 DOI: 10.1136/bmj.c4587. 20851841

[26] Ely JW, Osheroff JA, Ebell MH, Chambliss ML, Vinson DC, Stevermer JJ, Pifer EA Obstacles to answering doctors' questions about patient care with evidence: qualitative study. BMJ. 2002 3 23;324(7339):710. 11909789 10.1136/bmj.324.7339.710 PMC99056

[27] Cook DA, Sorensen KJ, Wilkinson JM, Berger RA Barriers and decisions when answering clinical questions at the point of care: a grounded theory study. JAMA Intern Med. 2013 11 25;173(21):1962–9. DOI: 10.1001/jamainternmed.2013.10103. 23979118

[28] Zwolsman S, te Pas E, Hooft L, Wieringa-de Waard M, van Dijk N Barriers to GPs' use of evidence-based medicine: a systematic review. Br J Gen Pract. 2012 7;62(600):e511–21. DOI: 10.3399/bjgp12X652382. 22781999 PMC3381277

[29] Cook DA, Sorensen KJ, Hersh W, Berger RA, Wilkinson JM Features of effective medical knowledge resources to support point of care learning: a focus group study. PLoS One. 2013;8(11):e80318 DOI: 10.1371/journal.pone.0080318. 24282535 PMC3840020

[30] Dunn K, Marshall JG, Wells AL, Backus JEB Examining the role of MEDLINE as a patient care information resource: an analysis of data from the Value of Libraries study. J Med Libr Assoc. 2017 10;105(4):336–46. DOI: 10.5195/jmla.2017.87. 28983197 PMC5624423

[31] Rübsam ML, Esch M, Baum E, Bösner S Diagnosing skin disease in primary care: a qualitative study of GPs' approaches. Fam Pract. 2015 10;32(5):591–5. DOI: 10.1093/fampra/cmv056. 26160890

[32] Swetter SM, Chang J, Shaub AR, Weinstock MA, Lewis ET, Asch SM Primary care-based skin cancer screening in a Veterans Affairs health care system. JAMA Dermatol. 2017 8 1;153(8):797–801. DOI: 10.1001/jamadermatol.2017.1324. 28593242 PMC5817609

[33] Barbieri JS, French B, Umscheid CA Uptake and impact of a clinical diagnostic decision support tool at an academic medical center. Diagnosis (Berlin, Germany). 2015 6 1;2(2):123–7. DOI: 10.1515/dx-2014-0058. 29540022

