# Supplementary material for: Barriers and facilitators to use of a clinical evidence technology in the management of skin problems in primary care: insights from mixed methods
Source: J Med Libr Assoc. 2020 Jul 1;108(3):428–39. doi: 10.5195/jmla.2020.787 (PMC7441913; doi:10.5195/jmla.2020.787)
Supplement: Supplementary file 1 — Appendix A: Survey of primary care providers post-cluster-randomized controlled trial [file jmla-108-3-428-s01.pdf]

Journal of the Medical Library Association

Supplemental content to
J Med Libr Assoc. Jul;108(3):dx.doi.org/10.5195/jmla.2020.787
www.jmla.mlanet.org
© Burke, Savard, Rubin, Littenberg 2020

Barriers and facilitators to use of a clinical evidence technology in the management of skin problems in primary care: insights from mixed methods

Marianne D. Burke, PhD, AHIP; Liliane B. Savard, DPT; Alan S. Rubin, MD; Benjamin Littenberg, MD

APPENDIX A

Survey of primary care providers post-cluster-randomized controlled trial

VisualDx user (intervention) arm questions

During the study, how many times did you refer to VisualDx?

In what percent of skin problem patients did you refer to it?

How useful was VisualDx in diagnosing and treating patients?
Scale 1–4: 1. Not at all 2. Occasionally 3. Usually 4. Always

How easy or difficult was it to find information you needed?
Scale 1–4: 1. Not at all 2. Occasionally 3. Usually 4. Always

Non-VisualDx user (control) arm questions

During the study participation period (ending July 31, 2016), did you refer to VisualDx?
Yes/No

Both arms questions

How often have you referred to VisualDx since the trial ended?

In the last month, how many times did you see a patient for a skin problem?

In last month, how many times did you look for additional information to support care for a patient skin problem.

In those times, what sources did you use?

- ❑ DynaMed
- ❑ Google
- ❑ Journal articles
- ❑ PubMed/MEDLINE
- ❑ UpToDate
- ❑ Textbooks (electronic or print)
- ❑ VisualDx
- ❑ Wikipedia
- ❑ Not sure
- ❑ Other

If other, please list.
