# Supplementary material for: Barriers and facilitators to use of a clinical evidence technology in the management of skin problems in primary care: insights from mixed methods
Source: J Med Libr Assoc. 2020 Jul 1;108(3):428–39. doi: 10.5195/jmla.2020.787 (PMC7441913; doi:10.5195/jmla.2020.787)
Supplement: Supplementary file 2 — Appendix B: Semi-structured interviews outline [file jmla-108-3-428-s02.pdf]

JMLA Journal of the Medical Library Association

SC
Supplemental content to
J Med Libr Assoc. Jul;108(3):dx.doi.org/10.5195/jmla.2020.787
www.jmla.mlanet.org
© Burke, Savard, Rubin, Littenberg 2020

Barriers and facilitators to use of a clinical evidence technology in the management of skin problems in primary care: insights from mixed methods

Marianne D. Burke, PhD, AHIP; Liliane B. Savard, DPT; Alan S. Rubin, MD; Benjamin Littenberg, MD

APPENDIX B
Semi-structured interviews outline

Please describe your experience of being (participating) in the study.

What was your experience like using VisualDx? (Your opinion of it?) [Prompt: how useful? hard/easy to use]

How did you usually access VisualDx? [Prompt: Such as device/network/portal, electronic health record (EHR), mobile]

How did you find your usual method? [Prompt hard/easy, fast, slow?]

What difference did VisualDx make in an aspect of patient care?

Could you describe a time when it did make a difference or perhaps when you hoped it would and it did not?

Do you use VisualDx now? What prompts you to use it or not?

What other information resources did you use then or do you use now for evidence for skin problems?

What else you would like to tell me about using clinical information resources relevant to dermatology or skin problems?
